# N-glycosylation in non-invasive and invasive intraductal papillary mucinous neoplasm

**DOI:** 10.1038/s41598-023-39220-4

**Published:** 2023-08-14

**Authors:** Heini Nieminen, Pirjo Nummela, Tero Satomaa, Annamari Heiskanen, Jukka O. Hiltunen, Tuomas Kaprio, Hanna Seppänen, Jaana Hagström, Harri Mustonen, Ari Ristimäki, Caj Haglund

**Affiliations:** 1 Department of Surgery, University of Helsinki and Helsinki University Hospital, P.O. Box 440, 00029 Helsinki, Finland; 2 Applied Tumor Genomics Research Program, Research Programs Unit, University of Helsinki and Helsinki University Hospital, Helsinki, Finland; 3 Glykos Finland Ltd, Helsinki, Finland; 4 Research Programs Unit, Translational Cancer Medicine Research Program, University of Helsinki, Helsinki, Finland; 5 Department of Pathology, HUSLAB, HUS Diagnostic Center, University of Helsinki and Helsinki University Hospital, Helsinki, Finland; 6 Departmentof Oral Pathology and Radiology, University of Turku, Turku, Finland

**Keywords:** Pancreatic cancer, Glycobiology

## Abstract

Intraductal papillary mucinous neoplasms (IPMNs), often found incidentally, are potentially malignant cystic tumors of the pancreas. Due to the precancerous nature, IPMNs lacking malignant features should be kept on surveillance. The follow-up relies on magnetic resonance imaging, which has a limited accuracy to define the high-risk patients. New diagnostic methods are thus needed to recognize IPMNs with malignant potential. Here, aberrantly expressed glycans constitute a promising new area of research. We compared the N-glycan profiles of non-invasive IPMN tissues (n = 10) and invasive IPMN tissues (n = 10) to those of non-neoplastic pancreatic controls (n = 5) by matrix-assisted laser desorption-ionization time-of-flight (MALDI-TOF) mass spectrometry. Both IPMN subgroups showed increased abundance of neutral composition H4N4 and decrease in H3N5F1, increase in sialylation, and decrease in sulfation, as compared to the controls. Furthermore, invasive IPMN showed an increase in terminal N-acetylhexosamine containing structure H4N5, and increase in acidic complex-type glycans, but decrease in their complex fucosylation and sulfation, as compared to the controls. In conclusion, the N-glycan profiles differed between healthy pancreatic tissue and non-invasive and invasive IPMNs. The unique glycans expressed in invasive IPMNs may offer interesting new options for diagnostics.

## Introduction

Widespread and increasing use of imaging modalities has led to an increasing detection rate of patients with asymptomatic pancreatic cysts. Intraductal papillary mucinous neoplasm (IPMN) is a common pancreatic cystic neoplasm and a typical incidental finding. IPMNs can be divided into three main types: the main duct (MD) type, the branch duct (BD) type, and a mixed type affecting both the main and branch ducts. IPMNs can be further divided into four subtypes according to histology and MUC expression: intestinal, pancreatobiliary, gastric, and oncocytic type. Over time, some of the IPMN tumors develop increasing grades of dysplasia and transform into an invasive carcinoma, invasive IPMN^1,2^. The risk of malignancy depends on the type and the histological subtype of IPMN. Indeed, main duct IPMNs have a higher risk to develop into carcinoma than the branch duct type IPMN^3^. The pancreatobiliary subtype, in turn, has the highest risk of undergoing malignant transformation and thus has the poorest outcome of the four histological subtypes^4,5^. Due to the malignant potential, IPMNs require follow-up as long as the patient is fit for major pancreatic surgery. Pancreatic surgery includes significant risk of complications and even mortality^6^ and the indications for surgery must be carefully considered. The prognosis after surgery depends on the stage of dysplasia and IPMN patients undergoing pancreatic surgery before malignant transformation have a good prognosis^7,8^. New prognostic markers indicating which tumors are likely to turn into malignant ones would thus be important, because high-risk patients could undergo earlier surgery and on the other hand, patients with tumors less likely to develop to an invasive IPMN could be offered a rationalized and less intensive surveillance.

Glycans are sugar assemblies covering the cell surface of all cells as protein- and lipid-linked glycoconjugates^9,10^. The major glycan types, N- and O-linked glycans differ from each other by the form of the covalent binding to the polypeptide part, being either nitrogen- or oxygen-linked, respectively. N-glycans are linked to the asparagine amino acids of polypeptides.^10^ Glycans play a critical role in normal physiological cell functions like adhesion, communication, and cell–cell recognition^9^. During malignant transformation, glycan profiles are altered. Changes in the branching of N-glycans, increased fucosylation and sialylation, and overexpression of truncated short O-glycans are typical alterations found in gastrointestinal cancers^11^. Also in pancreatic cancer increase in fucosylated and branched N-glycans has been reported, as well as upregulation of the sialyl Lewis antigens A and X, truncated O-glycans, and O-GlcNAcylation^12–14^.

The glycan profiles in IPMN serum samples have been studied before. Akimoto et al. measured the serum N-glycan profiles in IPMN patients and compared the changes with the patients’ clinical parameters^15^. They concluded that increased serum expression of fucosylated complex type glycans could be a potential marker for invasiveness. However, glycosylation of serum proteins does not directly reflect the glycosylation in the tumor tissue and pancreatic cancer-associated glycosylation changes have been reported in both IgG produced by B cells^16^ and acute phase proteins originating from the liver^17^. To our knowledge, the N-glycan profiles of IPMN tissue samples have not been reported earlier.

In this study, we determined the N-glycan profiles from paraffin embedded IPMN tumors with and without invasion, and healthy pancreatic tissues. Our aim was to compare the differences between healthy pancreatic tissue and IPMN, as well as between non-invasive IPMN and invasive IPMN.

## Materials and methods

### Patients

Experienced pathologists (A.R., J.H.) re-evaluated archived formalin-fixed and paraffin-embedded (FFPE) surgical specimens from all 98 patients who had been operated on for IPMN in the Helsinki University Hospital (HUH) between years 2000 and 2015 and found 11 representative IPMN tumor samples containing both invasive IPMN (carcinoma) and non-invasive IPMN areas within the same tumor. One patient had to be left out of the study due to technical problems with the samples. The healthy control tissue was obtained from five patients who had been operated on for neuroendocrine tumors between 2012 and 2015.

### Tissue specimens

The hematoxylin and eosin-stained slides were evaluated by two pathologists (A.R., J. H.), and the most representative areas were marked. The corresponding areas of the FFPE tissue blocks were then punched with a 3.0 mm puncher in order to gain tissue for glycan analysis. Images of representative hematoxylin and eosin stained slides are found in Supplementary Fig. S1. In addition to the tumor specimens, the healthy pancreas tissue samples were also evaluated by two pathologists and the selected area for punching was as far as possible from the neuroendocrine tumor. Xylene and descending ethanol series were used to deparaffinize the tissue samples as earlier described^18^.

This study was conducted in accordance with relevant guidelines and regulations: the use of archive tissue material was approved by the National Supervisory Authority of Welfare and Health (Valvira Dnro 10041/06.01.03.01/2012) and the study and the use of patient data was approved by the surgical ethical committee of HUH (Dnro HUS 226/E6/2006). Informed consent was obtained from the patients.

### N-glycan extraction and mass spectrometric analysis

N-glycan extraction and purification of neutral and acidic N-glycans, and the mass spectrometric analysis was performed as earlier described^18,19^. Briefly, the glycans were enzymatically detached by N-glycosidase F (PNGase F) digestion from the deparaffinized tissue specimens (Glyko; ProZyme Inc., Hayward, CA). After the digestion, the N-glycans were purified by solid-phase extraction in 96-well format. The Bruker Ultraflex III TOF/TOF instrument (Bruker Daltonics Inc, Bremen, Germany) was used to perform matrix-assisted laser desorption-ionization time-of-flight (MALDI-TOF) mass spectrometry to the glycan samples. The mass spectrometry detected acidic N-glycans in negative ion reflector mode primarily as [M− H]¯ ions and neutral N-glycans in positive ion reflector mode primarily as [M+ Na]^+^ ions. Satomaa et al.^20^ and Saarinen et al.^19^ have previously described how the raw data was processed into presented glycan profiles. All the MS data are presented in Supplementary Tables S1 and S2.

### Data analysis

N-glycan data was analyzed in three different study groups: non-neoplastic pancreatic controls, non-invasive IPMN, and invasive IPMN. The analyses were done separately for acidic and neutral glycan fractions, as well as for separate glycan structures (monosaccharide compositions) and structural/biosynthetic glycan classes.

### Statistics

False discovery rate (FDR)^21^ was used to adjust for multiple comparisons for all the *p*-values. *P*-values < 0.05 were considered as statistically significant and two-tailed tests were used. Principal component analysis (PCA) was performed using the Statistica software using default parameters for all glycans that showed significance by the Mann–Whitney U test.

### OPLS-DA

Orthogonal projections to latent structures discriminant analysis (OPLS-DA) modeling were performed using the ropls^31^ R package in R. For OPLS-DA modeling, both neutral and acidic proposed monosaccharide compositions with Mann–Whitney U test p-values of less than 0.1 were used in the same model. Permutation testing was employed to establish the significance of the R2Y and Q2Y values. Statistical analyses were carried out by using SPSS (IBM SPSS Statistics© version 24, International Business Machines Corp., NY, USA) and R (version 4.1.0, Foundation for Statistical Computing, Vienna, Austria).

## Results

### Patients

The 10 IPMN patients who were included in the study, four males and six females, had undergone surgery between 2002 and 2014. The median age was 72.5 (range 62–79). Two patients had main duct type, four had mixed type, and four had branch duct type IPMN (Table 1). Eight patients had pancreatobiliary subtype and two had intestinal subtype IPMN, and none had gastric or oncocytic type IPMN. The grade of dysplasia was low in six specimens, and high in four specimens (Table 1). One patient (number 2) had to be left out of the analysis because of technical problems with the tumor samples.

**Table 1:** Data of the ten IPMN patients of which both non-invasive (dysplastic) and invasive (carcinoma) areas were found from their surgical specimens.

Patient number	Gender	Age	Operation year	Ca19-9	IPMN maintype	IPMN Subtype	Dysplasia sample number	Grade of dysplasia	Cancer sample number	Cancer type	Neoadjuvant chemotherapy
1	male	73	2002	329	Main duct type	I	1,1	high grade	1,2	DAC	No
3	male	75	2004	758	Branch duct type	PB	3,1	low grade	3,2	DAC	No
4	female	62	2006	36	Branch duct type	PB	4,1	moderate	4,2	DAC	No
5	female	65	2009	14	Mixed type	PB	5,1	moderate	5,2	DAC	No
6	female	64	2011	62	Mixed type	PB	6,1	high grade	6,2	DAC	No
7	female	79	2011	76	Branch duct type	PB	7,1	high grade	7,2	DAC	Yes (gemcitabine)
8	female	79	2011	74	Mixed type	PB	8,1	low grade	8,2	DAC	No
9	male	70	2011	2	Branch duct type	PB	9,1	low grade	9,2	DAC	No
10	female	76	2012	122	Main duct type	I	10,1	high grade	10,2	DAC	No
11	male	72	2014	89	Mixed type	PB	11,1	moderate	11,2	DAC	No

Ca19-9 levels measured preoperatively.

*I* Intestinal, *PB* Pancreatobiliary, *DAC* ductal adenocarcinoma.

The healthy pancreatic tissue was obtained from five patients, four females and one male, who had been operated on for neuroendocrine tumors. The median age was 57 years (range 30–60).

### Neutral N-linked glycan profiles

**Figure 1:**
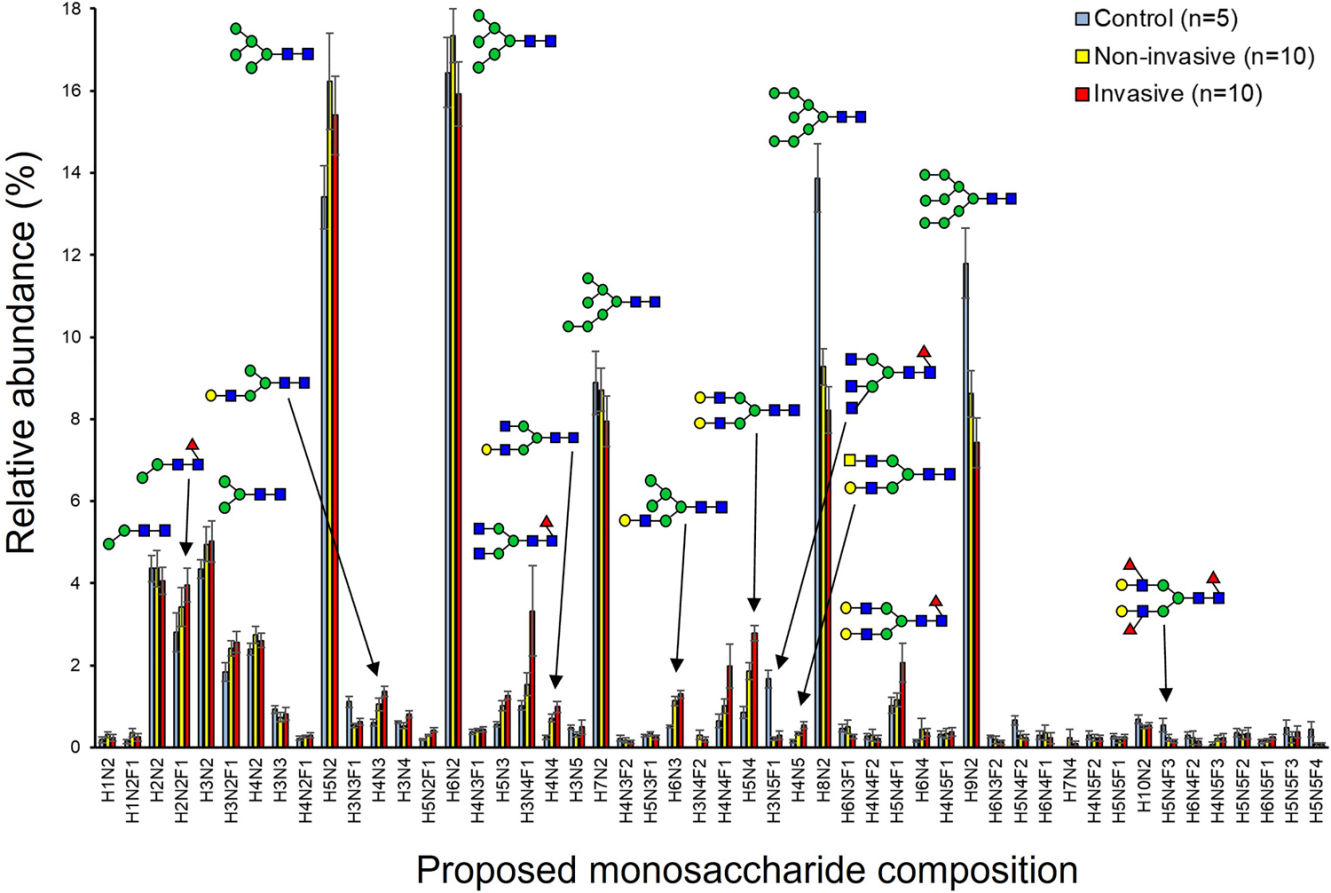
The 50 most abundant neutral N-glycan signals, neutral N-glycan profiles of non-invasive Intraductal papillary mucinous neoplasm (IPMN) (yellow bar, n = 10), invasive IPMN (red bars, n = 10), and healthy pancreatic tissue (blue bars, n = 5) as analyzed by mass spectrometry. The figure shows the relative abundance of the 50 most abundant N-glycan structures in the neutral N-glycan fraction in the order of increasing m/z value. The results are shown as mean ± SEM. Representative glycan structures are depicted by blue square (N-acetyl-D-glucosamine), green circle (D-mannose), yellow circle (D-galactose), and red triangle (L-fucose). *H* Hexose, *N* N-Acetylhexosamine, and *F* Deoxyhexose.

N-linked glycan profiles were analyzed by mass spectrometry from FFPE tissue specimens of 10 IPMN patients demonstrating both non-invasive IPMN and invasive IPMN (in the same tumor), as well as from five specimens of healthy pancreatic tissue. The neutral N-glycan profiles of both non-invasive and invasive IPMNs differed from those of healthy control samples. The relative abundances of the 50 most abundant neutral monosaccharide compositions are shown in Fig. 1 and the changes in the relative abundances are discussed below. The most abundant neutral glycans in all the groups were the high-mannose type structures H5N2 and H6N2 (small high-mannose), and H8N2 and H9N2 (large high-mannose), of which H8N2 was significantly decreased in both the non-invasive and invasive IPMN subgroups as compared to the controls (FDR-corrected *p*-values 0.032 and 0.015 for non-invasive and invasive, respectively) (Table 2). Also, H9N2 showed a trend of relative decrease in both the non-invasive and invasive IPMN subgroups compared to the controls. Therefore, both non-invasive and invasive IPMNs had smaller high-mannose type N-glycans when compared to the controls. Other neutral compositions significantly changed in both the IPMN subgroups as compared to the controls were the proposed non-fucosylated terminal N-acetylhexosamine (HexNac) containing structure H4N4 (increased in both, *p*-values 0.038 and 0.045, respectively), hybrid-type H6N3 (increased, *p*-values 0.038 and 0.015, respectively), and the fucosylated terminal HexNac containing H3N5F1 (decreased, *p*-values 0.032 and 0.025, respectively) (Table 2). Structures showing significant change (increase) only in invasive IPMN, as compared to the controls, included hybrid-type/monoantennary H4N3 (*p* = 0.025), fucosylated small high-mannose type H5N2F1 (*p* = 0.045), hybrid-type H5N3 (*p* = 0.015), complex-type H5N4 (*p* = 0.015), and the proposed terminal HexNac containing H4N5 (*p* = 0.015) (Table 3).

**Table 2:** Shared N-glycan alterations in non-invasive and invasive IPMNs as compared to the controls.

	Relative abundance		(%)	*p*-value*	
	Ctrl	Non-invasive	Invasive	Ctrl- Non-invasive	Ctrl-invasive
Neutral monosaccharide composition					
H4N4	0.3	0.7	1.0	0.038	0.045
H6N3	0.5	1.1	1.3	0.038	0.015
H3N5F1	1.7	0.2	0.3	0.032	0.025
H8N2	13.9	9.3	8.2	0.032	0.015
Neutral N-glycan class					
Monoantennary	35.9	23.1	22.5	0.035	0.010
Acidic monosaccharide composition					
H3N4P1	0.4	0.0	0.0	0.032	0.015
H5N3P1	2.7	0.2	0.0	0.032	0.015
H3N4F1P1	0.6	0.1	0.1	0.032	0.015
H6N3P1	7.4	1.5	0.8	0.038	0.015
H4N5F1P1	1.1	0.3	0.1	0.038	0.015
Acidic N-glycan class					
Sialylated	75.8	92.9	96.3	0.035	0.007
Monoantennary	31.8	49.5	51.2	0.035	0.020
Sulfate/phosphate	24.8	7.7	4.2	0.035	0.007
High-mannose type	1.8	0.3	0.3	0.035	0.026
Pauci-mannose type	1.0	0.2	0.1	0.035	0.012
Sulfate/phosphate in hybrid-type	44.5	13.8	9.5	0.035	0.007

*Ctrl* healthy control.

*H* Hexose, *N* N-Acetylhexosamine, *F* Deoxyhexose (fucose), *P* Acid ester (sulfate/phosphate).

*Mann–Whitney U test with Benjamini–Hochberg false discovery rate correction.

**Table 3:** Significant changes detected only in the N-glycan profiles of invasive IPMNs as compared to the controls.

	Relative abundance		(%)	*p*-value**
	Ctrl	Non-invasive	Invasive	Ctrl-invasive
Neutral monosaccharide composition				
H4N3	0.6	1.1	1.4	0.025
H5N2F1	0.2	0.3	0.4	0.045
H5N3	0.6	1.0	1.3	0.015
H5N4	0.9	1.9*	2.8	0.015
H4N5	0.1	0.3	0.6	0.015
Neutral N-glycan class				
4 HexNAc	6.9	9.6	13.7	0.012
Small high-mannose type	20.8	26.9	28.4	0.010
Fucosylation in high-mannose type	0.6	0.8	1.2	0.012
Fucosylation in hybrid-type	52.9	39.0	30.2	0.007
Fucosylation in complex-type	76.7	59.1*	60.2	0.026
Acidic monosaccharide composition				
H6N2P1	0.9	0.2*	0.1	0.015
H3N5F1P1	0.5	0.0*	0.0	0.047
H7N3P1	1.2	0.3	0.3	0.047
H5N4F1P1	1.0	0.5	0.4	0.015
H3N6F1P1	0.6	0.0*	0.0	0.047
H4N5F2P1	1.1	0.3*	0.2	0.015
Acidic N-glycan class				
2 HexNAc	2.8	0.5*	0.4	0.012
3 HexNAc	26.2	18.8	14.5	0.012
4 HexNAc	56.4	64.7	70.1	0.026
Complex-type	71.0	80.8	85.1	0.012
Sialylation in complex-type	86.7	94.9*	97.5	0.007
Complex fucosylation in hybrid-type	1.2	3.3	0.2	0.039
Complex fucosylation in complex-type	9.6	9.6	5.0	0.026
Sulfate/phosphate in oligo-mannose type	84.0	56.4	26.3	0.026
Sulfate/phosphate in complex-type	13.9	5.8	3.1	0.010

*Ctrl* healthy control.

*H* Hexose, *N* N-Acetylhexosamine, *F* Deoxyhexose (fucose), *P* Acid ester (sulfate/phosphate), *HexNac* N-Acetylhexosamine.

*Marginally significant in non-invasive IPMN (0.05 < *p* < 0.09).

**Mann–Whitney U test with Benjamini–Hochberg false discovery rate correction.

**Figure 2:**
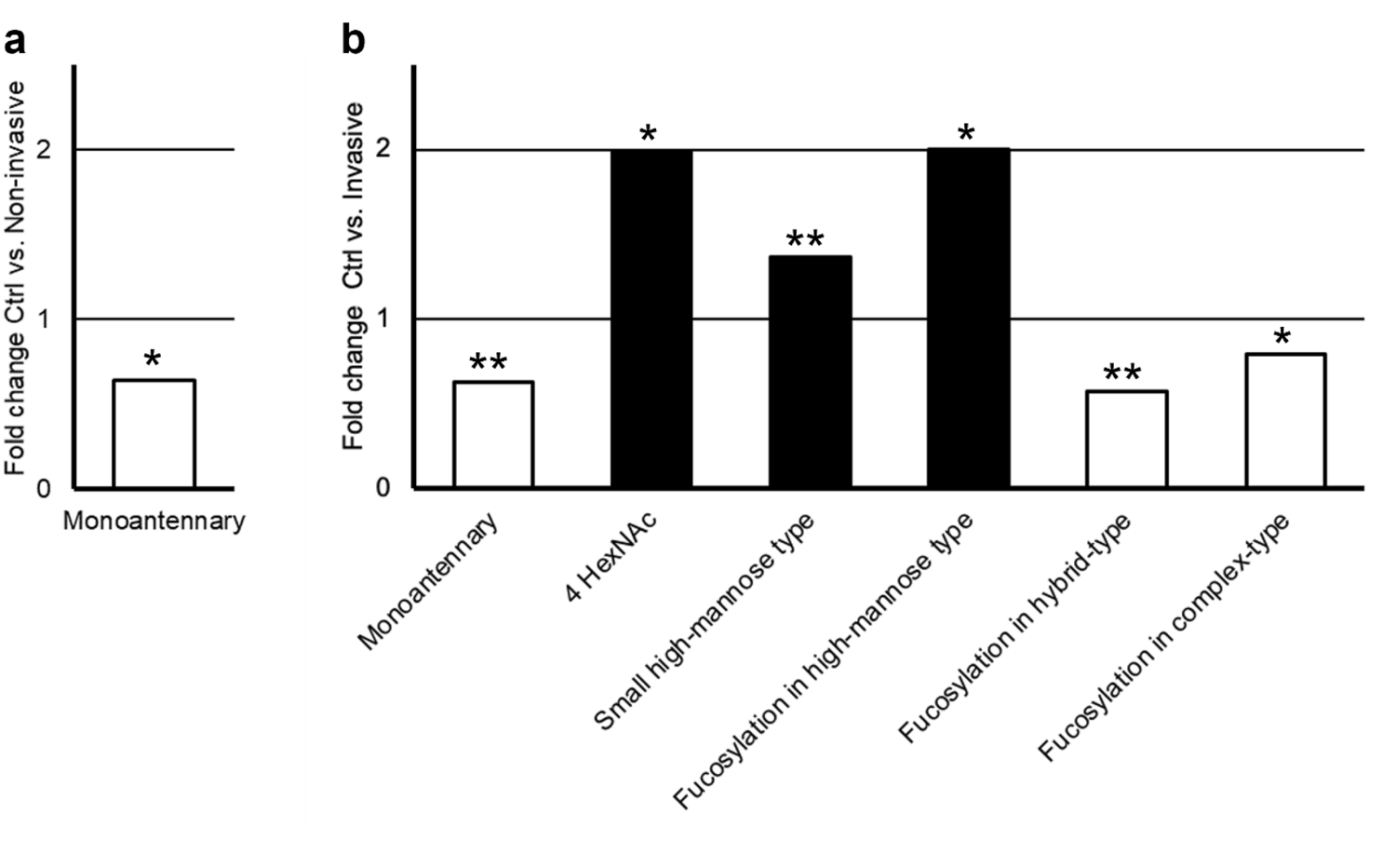
Differences in neutral N-glycosylation between (**A**) healthy pancreatic tissue controls (Crtl) and non-invasive Intraductal papillary mucinous neoplasm (IPMN), and (**B**) healthy pancreatic tissue and invasive IPMN. Fold change of the mean relative intensity of structural glycan classes between different tissues. Statistical analysis by the Mann–Whitney U test: * (*p* < 0.05), ** (*p* ≤ 0.01) FDR-adjusted *p*-values. Black bars = increase in IPMN, white bars = decrease in IPMN.

When the neutral monosaccharide compositions were assigned to structural/biosynthetic glycan classes, only monoantennary glycan class was found to be significantly changed in both the non-invasive and invasive IPMN subgroups as compared to the controls (decreased, FDR-corrected *p*-values 0.035 and 0.010, respectively) (Table 2). However, in invasive IPMNs, as compared to the controls, several additional glycan classes were found to show significant change. These classes included four HexNAcs containing structures (increased, *p* = 0.012), small high-mannose type (increased, *p* = 0.010), fucosylation in high-mannose type (increased, *p* = 0.012), as well as fucosylation in hybrid-type and complex-type glycans (decreased, *p*-values 0.007 and 0.026, respectively) (Table 3). The fold change values of these neutral N-glycan alterations are shown in Fig. 2.

### Acidic N-linked glycan profiles

**Figure 3:**
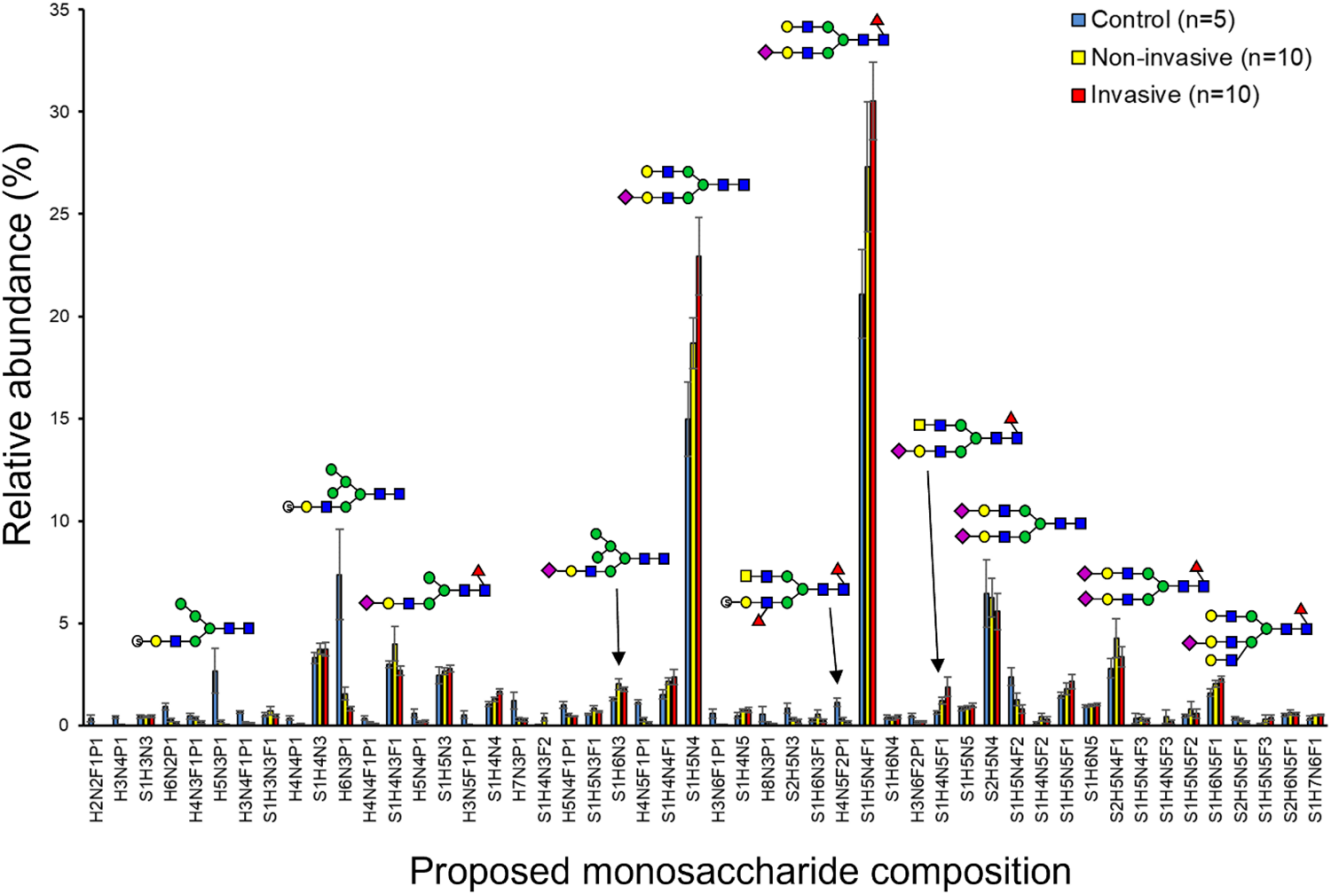
The 50 most abundant acidic N-glycan signals, acidic N-glycan profiles of non-invasive Intraductal papillary mucinous neoplasm (IPMN) (yellow bar, n = 10), invasive IPMN (red bars, n = 10), and healthy pancreatic tissue (blue bars, n = 5) as analyzed by mass spectrometry. The figure shows the relative abundance of the 50 most abundant acidic N-glycan structures in the order of increasing m/z value. The results are shown as mean ± SEM. Representative glycan structures are depicted by blue square (N-acetyl-D-glucosamine), green circle (D-mannose), yellow circle (D-galactose), red triangle (L-fucose), and purple diamond (N-acetylneuraminic acid i.e. sialic acid). *H* Hexose, *N* N-Acetylhexosamine, *F* Deoxyhexose, *S* Sialic Acid, and *P* Acid ester.

Acidic N-glycan profiles, consisting of glycans modified by sialylation or acid esters (most often sulfate, also phosphate), were analyzed mass spectrometrically from non-invasive IPMN, invasive IPMN, as well as healthy control tissue specimens. The acidic N-glycan profiles were different between the three sample groups. The relative abundances of the 50 most abundant acidic N-glycans are shown in Fig. 3 and the changes in the relative abundances are discussed below. The most abundant glycans in the acidic glycan profiles of all the sample groups were the sialylated complex-type N-glycans S1H5N4 and S1H5N4F1. Acidic compositions showing significant change in both the non-invasive and invasive IPMN subgroups as compared to healthy controls included the decreased acid ester modified (sulfated/phosphorylated) structures H3N4P1 (FDR-corrected p-values 0.032 and 0.015, respectively), H5N3P1 (*p*-values 0.032 and 0.015, respectively), H3N4F1P1 (*p*-values 0.032 and 0.015, respectively), H6N3P1 (*p*-values 0.038 and 0.015, respectively), and H4N5F1P1 (*p*-values 0.038 and 0.015, respectively) (Table 2). Structures showing significant change (decrease) only in invasive IPMN, as compared to the controls, included the sulfated structures H6N2P1 (*p* = 0.015), H3N5F1P1 (*p* = 0.047), H7N3P1 (*p* = 0.047), H5N4F1P1 (*p* = 0.015), H3N6F1P1 (*p* = 0.047), and H4N5F2P1 (*p* = 0.015) (Table 3).

**Figure 4:**
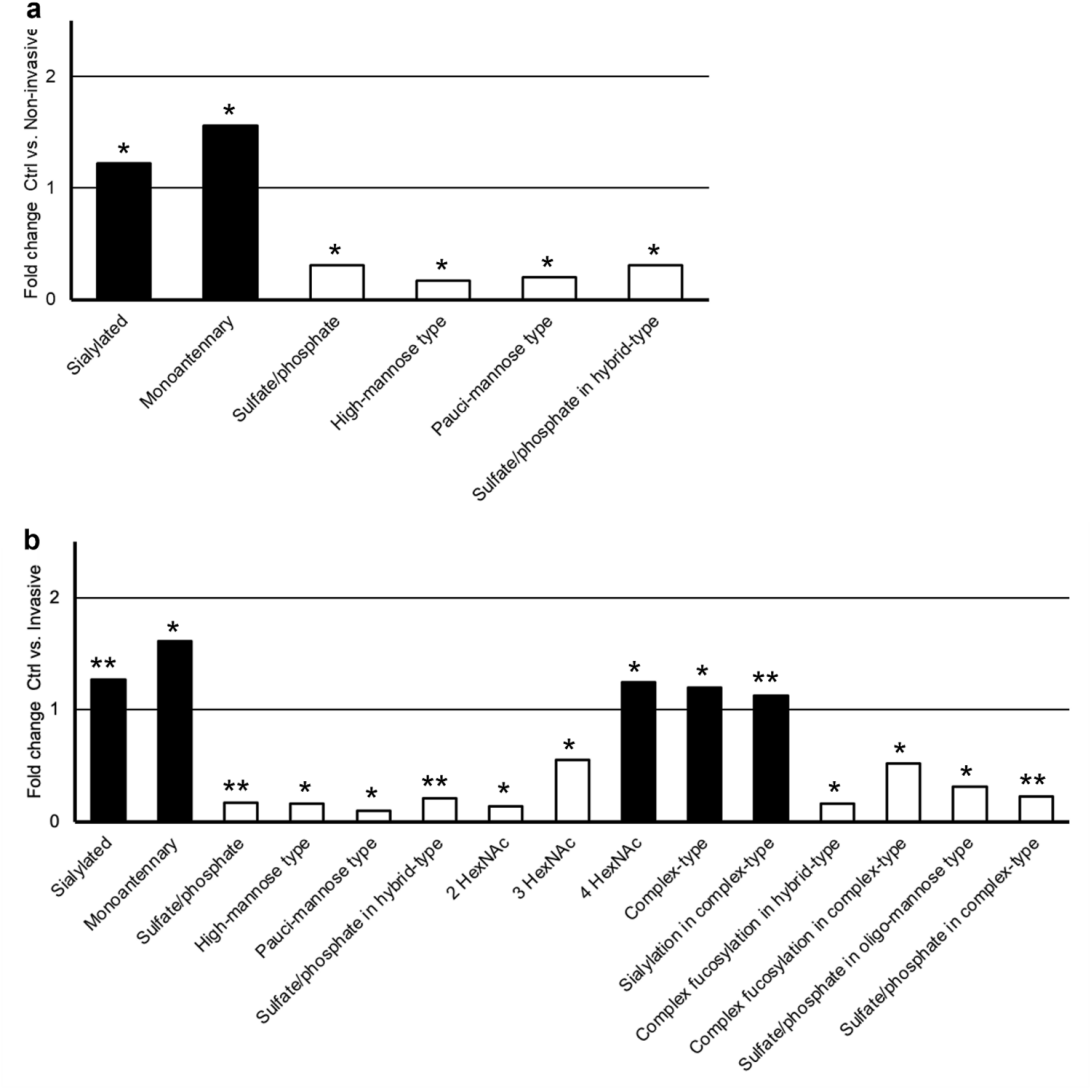
Differences in acidic N-glycosylation between (**A**) healthy pancreatic tissue (Ctrl) and non-invasive Intraductal papillary mucinous neoplasm (IPMN), and (**B**) healthy pancreatic tissue and invasive IPMN. Fold change of the mean relative intensity of structural glycan classes between different tissues. Statistical analysis by the Mann–Whitney U test: * (*p* < 0.05), ** (*p* ≤ 0.01). Black bars = increase in IPMN, white bars = decrease in IPMN.

When the acidic monosaccharide compositions were assigned to structural/biosynthetic glycan classes, several glycan classes were found to be changed in both the non-invasive and invasive IPMN subgroups as compared to the controls: sialylated glycans (increased, FDR-corrected p-values 0.035 and 0.007, respectively), monoantennary (increased, *p*-values 0.035 and 0.020), sulfated/phosphorylated glycans (decreased, *p*-values 0.035 and 0.007), high-mannose type (decreased, *p*-values 0.035 and 0.026), pauci-mannose type (decreased, *p*-values 0.035 and 0.012), and acid ester modified (sulfated/phosphorylated) hybrid-type glycans (decreased, *p*-values 0.035 and 0.007) (Table 2). Acidic glycan classes showing significant increase only in invasive IPMNs, as compared to the controls, in turn, included four HexNAcs containing glycans (*p* = 0.026), complex-type glycans (*p* = 0.012), and sialylated complex-type glycans (*p* = 0.007). Further, acidic glycan glasses showing decrease only in invasive IPMNs, as compared to the controls, included two or three HexNAcs containing structures (indicating oligo-mannose and hybrid-type structures, respectively; *p* = 0.012 for both), complex fucosylation in hybrid-type and complex-type glycans (*p* < 0.04 for both), as well as sulfate/phosphate in oligo-mannose and complex-type glycans (*p* < 0.03 for both) (Table 3). The fold change values of these acidic N-glycan alterations are shown in Fig. 4.

### Principal component analysis of differently expressed neutral and acidic glycans

**Figure 5:**
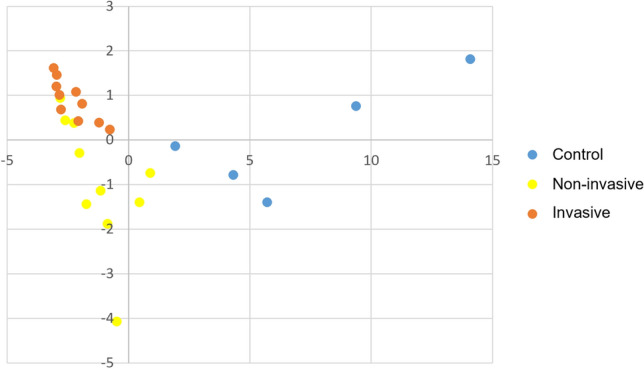
Principal component analysis (PCA) of Intraductal papillary mucinous neoplasm (IPMN) shows clustering of the non-invasive (yellow) and invasive IPMN (red) samples. Healthy pancreatic tissue is shown in blue. Figure shows acidic N-glycan significant signals (glycans that showed significance by the Mann–Whitney U test) PLS-DA (partial least squares with discriminant analysis), where x-axis shows t1 and y-axis shows t2.

The differences between the three groups, non-invasive IPMN, invasive IPMN and healthy pancreatic tissue, were also visualized with the principal components analysis (PCA) (Fig. 5) where the healthy control specimens cluster together and the IPMN specimens together. The analysis showed some overlap of the invasive and non-invasive specimens, but the control specimens were separate.

### OPLS-DA analysis

**Figure 6:**
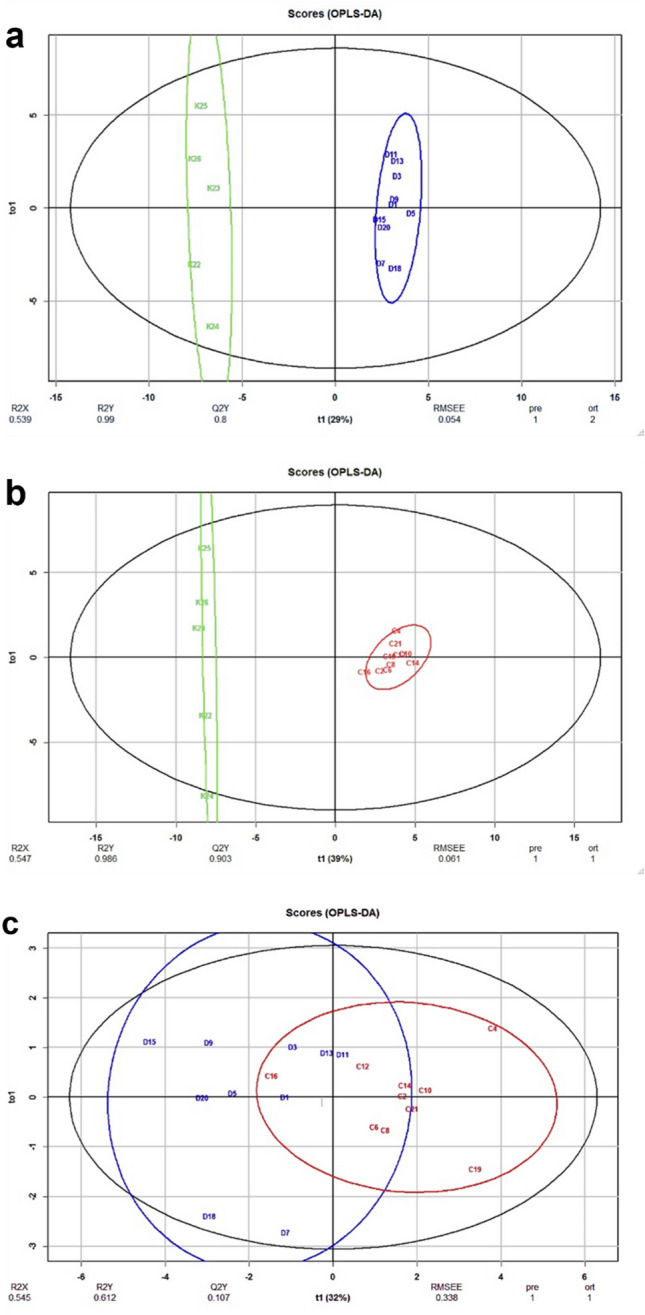
Multivariate (OPLS-DA) modelling of healthy controls, non-invasive IPMN and invasive IPMN samples by neutral and acidic glycans. Score plot shows how well the predictive component (t1) of multivariate model can separate the samples from each other. (**A**) Scoreplot of healthy pancreatic tissue vs. non-invasive IPMN (the predictive component (t1) on the x-axis and the orthogonal component (to1)on the y-axis, permutation test for Q2Y *p* = 0.002 and for R2Y *p* = 0.002), (**B**) Score plot of healthy pancreatic tissue vs. invasive IPMN (permutation test for Q2Y *p* = 0.001 and for R2Y *p* = 0.001), C) Score plot of non-invasive IPMN vs. invasive IPMN (permutation test for Q2Y *p* = 0.111 and for R2Y *p* = 0.101). R2X and R2Y are proportion of predictor/response variation explained by the full model, respectively. Q2Y is predictive performance of the model, RMSEE is root mean squared error of estimation, pre is the number of predictive components, ort is number of orthogonal components. Both neutral and acidic proposed monosaccharide compositions with the Mann–Whitney U test *p*-values of less than 0.1 were used in the models. Black ellipse is the 95% confidence interval for the model and colored (green = healthy, blue = non-invasive and red = invasive) ellipses are the 95% of multivariate normal distribution for each group of samples. The percentage of response variance explained by the predictor component (t1) is indicated in parentheses.

We also did an OPLS-DA analysis (Orthogonal projections to latent structures discriminant analysis), which in addition to PCA, visualizes the differences between the sample groups, and shows how well the multivariate model can separate the samples from each other. As seen in Fig. 6A and B, the control samples were separated from non-invasive IPMN or from invasive IPMN samples with very good quality or fit of model (R2Y = 0.990 and 0.986, for non-invasive and invasive IPMN, respectively), and with fairly good separation between classes (Q2Y = 0.8 and 0.903, respectively). However, the non-invasive and invasive samples could not be separated, and the predictive performance of the model was very poor. We also performed permutation testing to avoid overfitting and assess the statistical significance of the models shown in a Supplementary Fig. S2. According to permutation testing the OPLS-DA analyses with control versus non-invasive IPMN or invasive IPMN overfitting was not a problem, and these models are adequate. However, the model comparing non-invasive IPMN and invasive IPMN was poor in all respects.

## Discussion

In our study, we used MALDI-TOF MS profiling to demonstrate that the neutral and acidic N-glycan profiles of non-invasive (dysplastic) IPMN tissues and invasive IPMNs differ from those of healthy pancreatic tissues. The serum N-glycan profiles of IPMNs have been studied before^15^, but, to our knowledge, this study is the first one to analyze the glycan profiles of IPMN tissues.

As compared to the healthy controls, non-invasive and invasive IPMNs showed several shared N-glycosylation alterations, whereas some alterations only reached significance in invasive IPMNs. Among the neutral N-glycan structural classes, only hybrid-type/monoantennary glycans were significantly changed, decreased, in both the IPMN subgroups as compared to the controls, whereas several glycosylation changes were detected between the controls and invasive IPMNs. These changes included increase in four HexNAcs containing structures (indicating complex-type structures) and small high-mannose type structures, as well as fucosylation in high-mannose type structures. Fucosylation in hybrid-type and complex-type structures was, in turn, decreased. Kaprio et al. have published similar results when comparing rectal adenomas and carcinomas^18^, demonstrating small high-mannose type, fucosylated paucimannose type, and non-sialylated complex-type glycans, among others, to be characteristic for carcinomas. Balog et al. have reported fucosylated paucimannose and high-mannose N-glycans in colorectal carcinoma^22^ and Leijon et al. have detected fucosylated paucimannose N-glycans in metastasized pheochromocytomas and paragangliomas^23^. The group of fucosylated oligomannose N-glycans have thus recently been associated with several cancer types, while their origin and function remain unknown. Increased sialylation and fucosylation are two of the glycosylation irregularities also found in pancreatic cancer^24^.

Of the separate neutral N-glycan structures showing changed relative abundance in both the IPMN subgroups, very interesting ones are the non-fucosylated terminal HexNAc containing structure H4N4 (increased) and the fucosylated terminal HexNAc containing H3N5F1 (decreased). Further, the non-fucosylated terminal HexNAc containing H4N5 was increased in invasive IPMNs as compared to the controls. A putative terminal HexNAc structure is terminal GlcNAc that has indeed been associated with carcinoma tissue before^19,25^. In the study of Mann et al., H4N5 structure was more abundant in IPMN cyst fluid than in those of pancreatic serous cystadenomas, mucinous cystic neoplasms, or pseudocysts^26^. Similar to the previous publications, also in this study we observed terminal HexNAc both in healthy control tissues and in invasive and non-invasive IPMN samples, but the abundance of terminal HexNAc was significantly higher in the IPMN samples. Terminal GlcNAc has also been shown to be more abundant in metastasized pheochromocytomas and paragangliomas than in non-metastasized ones^23^. In the same publication^23^, fucosylated paucimannose type glycans were more abundant in malignant tumors similarly as in our study. The fucosylated structure H3N5F1, in turn, was decreased in both IPMN subtypes. The present results therefore indicate less abundant fucosylation of many N-glycan structures in the non-invasive and invasive IPMN tissues than in the control tissue samples. This is in contrast to the results of Akimoto et al. from IPMN patient sera, where several fucosylated glycan structures were elevated^15^, suggesting differential glycosylation in tissues and secreted glycoproteins in IPMN.

Further differences to the reported IPMN serum glycosylation were seen in the acidic N-glycan profiles. We saw a significant decrease in acid ester modification and increase in sialylation in all the IPMN tissue samples. Of the possible acid ester modifications, sulfation and phosphorylation, sulfation is common in the normal epithelium of the digestive tract^27,28^. Sialylation, on the other hand, is one of the main changes seen in gastrointestinal cancers and plays an important role in cell adhesion, signaling and cellular recognition^11^. Significant changes in relative abundance of acidic glycans detected only in invasive IPMNs included increased abundance of complex-type glycans, as well as increased sialylation, but decreased sulfation and complex fucosylation of these complex-type glycan structures. In the serum N-glycan profiles in IPMN, Akimoto et al.^15^ found that the expression of tri- and tetra-antennary glycans with fucose residues correlated with malignant cytology and risk features such as enhancing solid components and mural nodules. In their study, a high serum concentration of S3H6N5F1 (m/z 3195), a tri-antennary glycan with sialic acid and fucose residues, correlated with invasiveness of the IPMN. Also, high levels of sialylated and fucosylated structures S2H6N5F1 (m/z 2890) and S3H6N5F2 (m/z 3341) were risk factors for invasive IPMN in univariate analysis. In our study, the abundance of trisialylated S3 glycans was negligible. Additionally, the glycan S2H6N5F1 showed only < 1% abundance in all the specimens. Since Akimoto et al. studied the glycan changes from serum and we from tissues, the results are interesting to compare. It is difficult to conclude which glycans in the circulation originate from the tumor tissue itself and which from other tissues, especially the liver.

The challenge in IPMN patients treatment is the large number of patients under surveillance and the clinical difficulties in defining the risk of malignancy based only on MRI imaging, or in difficult cases, combined with invasive tissue sampling by endoscopic ultrasound or by ultrasound guided fine needle aspiration biopsy. In our study we used paraffin-embedded surgical tumor samples and found several N-glycosylation changes, as compared to healthy tissues, which were more prevalent in invasive IPMNs than in non-invasive dysplastic IPMNs. Indeed, all the alterations could be detected from both IPMN sample types, but some of them only reached significance in the malignant phase. We compared also the non-invasive dysplastic and invasive IPMNs but there were no statistically significant differences between the two groups (data not shown). In the future, based on the new knowledge on the N-glycan profiles in IPMN, it would be desirable to develop non-invasive methods to characterize IPMNs and the grade of dysplasia, and thereby to define the patients that benefit from surgery.

Finding and recognizing N-glycans specific for invasive IPMNs could open up also new therapeutic possibilities. Cancer specific N-glycans could be used as targets for tumor cell-surface protein specific antibodies in antibody drug conjugates (ADCs). In ADCs, a highly potent cytotoxic drug is attached to a specific antibody, leading to an extremely selective local cytotoxic effect^29^. Anti-glycan ADCs against glycan structures of pancreatic carcinomas such as STn and Globo H have been described^30,31^. Prendergast et al.^30^ showed that reduction in tumor volumes in in vivo models and tumor growth inhibition was seen in multiple cancer types in the study with Globo H^31^.

We acknowledge some limitations of our study. Although the number of IPMN patients in surveillance is increasing, the number of patients undergoing resection for IPMN is limited. Further, among the surgical specimens it is extremely demanding to find areas of non-invasive dysplasia and invasive IPMN in the same surgical specimen, and this challenge limited our sample size to only 10 patients. Our results therefore need to be confirmed in a larger study. Further, we had eight patients with pancreatobiliary subtype and two patients with intestinal subtype IPMN in our patient series. We did not have any patients with gastric or oncocytic subtype IPMN. This is in line with the behavior of these histological subtypes, as pancreatobiliary subtype is known to have a more aggressive biology and more malignant behavior than the other subtypes^4,5^ and this might explain why in our operated IPMN series pancreatobiliary subtype was emphasized.

In addition, the choice of using control pancreatic tissue from patients with neuroendocrine tumors can be disputed. The microscopically healthy pancreatic tissues were from patients undergoing surgery for a neuroendocrine tumor, with the healthy pancreatic tissue specimens taken as far away from the tumor as possible but it can be argued whether the pancreas with any kind of tumor is totally healthy. However, we saw no other possibility for obtaining healthy pancreatic tissue for control purposes, as totally healthy pancreases are not resected or removed. In our opinion, pancreatic tissue from IPMN patients would have been a poorer solution. Indeed, it’s known that in main duct type IPMNs, the uninvolved pancreas often reflects changes of chronic obstructive pancreatitis^32^. The third issue is the fact that IPMN tumors originate from the ductal epithelium and the healthy pancreatic tissue is mainly composed of acinary glands. It can be argued that some of the differences we observed between the IPMN samples and healthy controls came from differences between ductal and acinar structures.

IPMN, a pancreatic tumor, is resembling another slowly progressing mucinous tumor, the appendix-originating pseudomyxoma peritonei (PMP), whose N-glycan profiles we have analyzed earlier^19,33^. Even if the mutation profiles of IPMN and PMP are very similar, with prevalent KRAS and GNAS mutations^34,35^, their N-glycan profiles show notable differences. Of the shared glycosylation alterations, both invasive IPMNs and PMP tumors show decrease in the relative abundance of acidic hybrid-type structures and a concomitant increase in the acidic complex-type structures as compared to their respective normal controls. Sialylation and fucosylation, the two frequent glycosylation alterations in cancer, however, differ between IPMN and PMP. Sialylation is increased in both non-invasive and invasive IPMNs, but in PMP tumors the abundance remains unchanged. Fucosylation in neutral hybrid- and complex-type structures, in turn, is increased in PMP but decreased in invasive IPMN. Further, complex fucosylation (terminal fucosylation) is increased in neutral N-glycans and acidic hybrid-type structures of PMPs but doesn’t change in neutral N-glycans of IPMNs and, conversely, decrease in the acidic hybrid-type structures of invasive IPMNs.

## Conclusion

In our study, the N-glycan profiles showed differences between healthy pancreatic tissue and non-invasive IPMN or invasive IPMN. Most importantly, some of the changes were significant in invasive IPMNs, the carcinoma phase, only. Thereby, invasiveness associated glycans in IPMN may offer interesting new options for diagnostics and as potential therapeutic targets. Further studies on larger patient series are however needed.

### Supplementary Information

Supplementary Table S1.

Supplementary Table S2.

Supplementary Figure S1.

Supplementary Figure S2.

## Data Availability

The datasets generated and analyzed during the current study are available from the corresponding author on a reasonable request.

## Abbreviations

ADC: Antibody drug conjugate
BD: Branch duct
FDR: False discovery rate
FFPE: Formalin-fixed and paraffin-embedded
GlcNAc: N-acetyl-D-glucosamine
HexNAc: N-acetylhexosamine
HUH: Helsinki University Hospital
IPMN: Intraductal papillary mucinous neoplasm
MALDI-TOF MS: Matrix-assisted laser desorption-ionization time-of-flight mass spectrometry
MD: Main duct
MRI: Magnetic resonance imaging
MS: Mass spectrometry
OPLS-DA: Orthogonal projections to latent structures discriminant analysis
PCA: Principal components analysis
PNGase F: N-glycosidase F
