# Supplementary material for: N-glycosylation in non-invasive and invasive intraductal papillary mucinous neoplasm
Source: Sci Rep. 2023 Aug 14;13:13191. doi: 10.1038/s41598-023-39220-4 (PMC10425445; doi:10.1038/s41598-023-39220-4)
Supplement: Supplementary file 3 — Supplementary Figure S1. [file 41598_2023_39220_MOESM3_ESM.pdf]

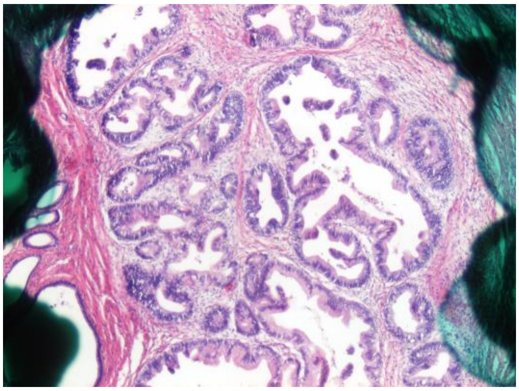

A1. Sample 4.1 Non-invasive

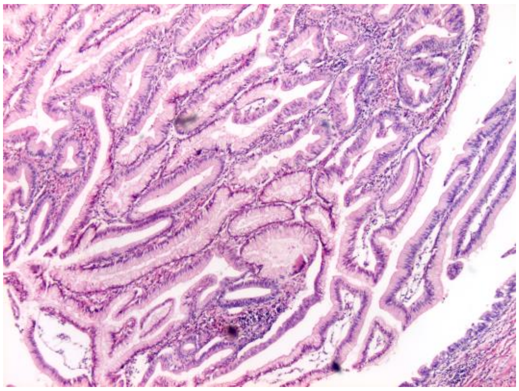

A2. Sample 4.2 Invasive

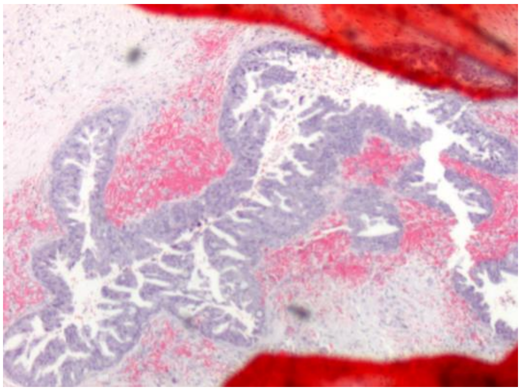

B1. Sample 7.1 Non-invasive

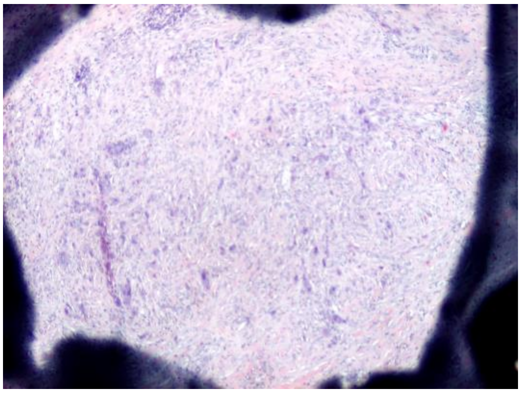

B2. Sample 7.2 Invasive

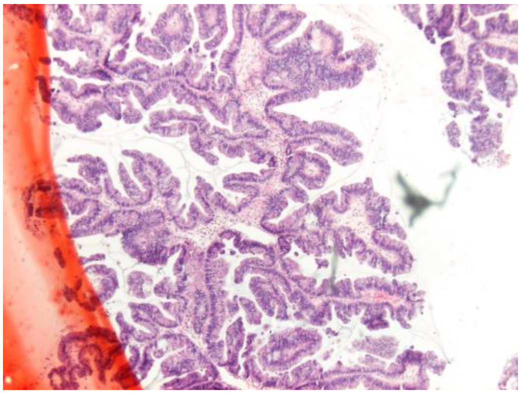

C1. Sample 10.1 Non-invasive

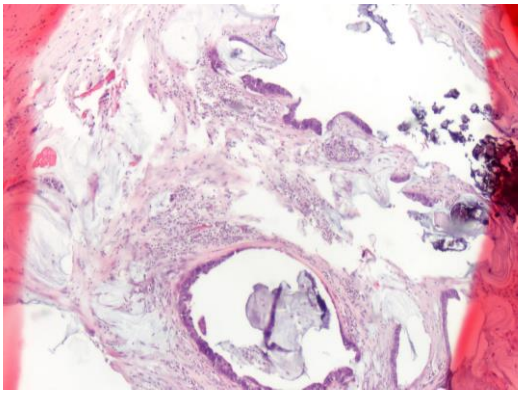

C2. Sample 10.2 Invasive

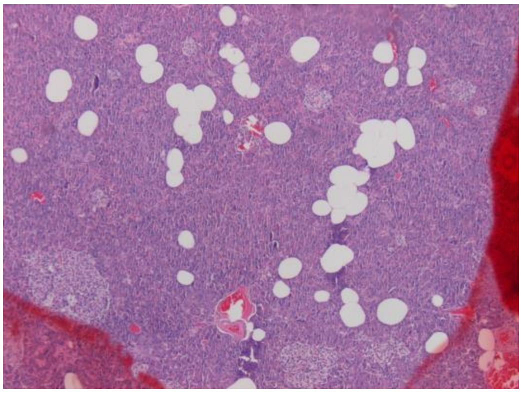

D. Sample Healthy control (3.)

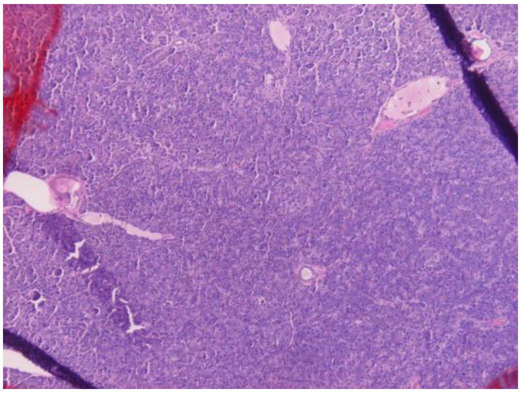

E. Sample Healthy control (4.)

Supplementary Figure S1. Images of representative hematoxylin and eosin stained slides of IPMN samples and healthy controls. **A1 and A2)** Patient number 4, **B1 and B2)** Patient number 7, **C1 and C2)** Patient number 10, **D)** Healthy control number 3., **E)** Healthy control number 4
