# Supplementary material for: N-glycosylation in non-invasive and invasive intraductal papillary mucinous neoplasm
Source: Sci Rep. 2023 Aug 14;13:13191. doi: 10.1038/s41598-023-39220-4 (PMC10425445; doi:10.1038/s41598-023-39220-4)
Supplement: Supplementary file 4 — Supplementary Figure S2. [file 41598_2023_39220_MOESM4_ESM.pdf]

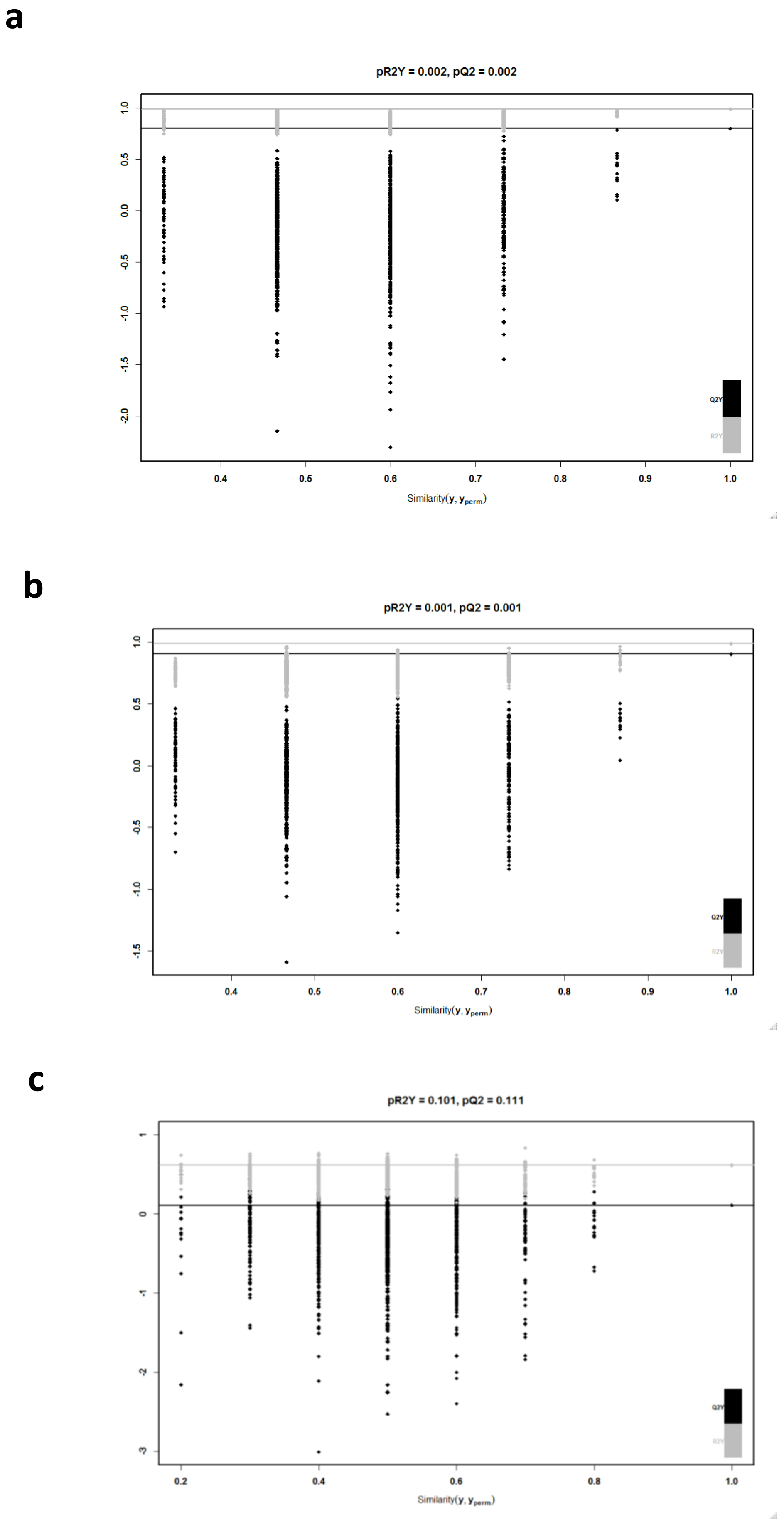

Supplementary Figure S2. Permutation testing of significance of R2Y and Q2Y values. A) Model between healthy tissues and non-invasive IPMN, B) Model between healthy tissues and invasive IPMN, C) Model between non-invasive and invasive IPMN. One thousand permutations were performed.
